# Novel pH-Sensitive Cyclic Peptides

**DOI:** 10.1038/srep31322

**Published:** 2016-08-12

**Authors:** Dhammika Weerakkody, Anna Moshnikova, Naglaa Salem El-Sayed, Ramona-Cosmina Adochite, Gregory Slaybaugh, Jovana Golijanin, Rakesh K. Tiwari, Oleg A. Andreev, Keykavous Parang, Yana K. Reshetnyak

**Affiliations:** 1 Department of Physics, University of Rhode Island, Kingston, RI 02881, US; 2 Department of Biomedical and Pharmaceutical Sciences, Chapman University School of Pharmacy, 9401 Jeronimo Road, Irvine, CA 92618, US; 3 Department of Biomedical and Pharmaceutical Sciences, College of Pharmacy, University of Rhode Island, 7 Greenhouse Road Kingston, RI 02881, US; 4 Cellulose and Paper Department, National Research Center, Dokki 12622, Cairo, Egypt

## Abstract

A series of cyclic peptides containing a number of tryptophan (W) and glutamic acid (E) residues were synthesized and evaluated as pH-sensitive agents for targeting of acidic tissue and pH-dependent cytoplasmic delivery of molecules. Biophysical studies revealed the molecular mechanism of peptides action and localization within the lipid bilayer of the membrane at high and low pHs. The symmetric, c[(WE)_4_WC], and asymmetric, c[E_4_W_5_C], cyclic peptides translocated amanitin, a polar cargo molecule of similar size, across the lipid bilayer and induced cell death in a pH- and concentration-dependent manner. Fluorescently-labelled peptides were evaluated for targeting of acidic 4T1 mammary tumors in mice. The highest tumor to muscle ratio (5.6) was established for asymmetric cyclic peptide, c[E_4_W_5_C], at 24 hours after intravenous administration. pH-insensitive cyclic peptide c[R_4_W_5_C], where glutamic acid residues (E) were replaced by positively charged arginine residues (R), did not exhibit tumor targeting. We have introduced a novel class of cyclic peptides, which can be utilized as a new pH-sensitive tool in investigation or targeting of acidic tissue.

Tissue acidity is linked to various pathological states such as ischemia, tumor, inflammation, arthritis, infection, atherosclerosis and others^1^,^2^,^3^. Tumor progression and development is associated with acidosis^4^,^5^,^6^. Extracellular acidity is established already at early stages of tumor development, during the avascular phase of carcinoma *in situ*. As tumor continues to grow, acidosis is increasing due to the poor blood perfusion, switch of cancer cells to the glycolytic mechanism of energy production even in the presence of oxygen and overexpression of carbonic anhydrases (CA)^7^,^8^. Adaptations to the highly acidic microenvironment are critical steps in the transition from an avascular pre-invasive tumor to a malignant invasive carcinoma. Thus, targeting of acidity might serve as a predictive marker for tumor invasiveness and disease development. pH is especially lower in the vicinity of the membrane of cancer cells due to the work of proton pumps and CAIX/CAXII^9^. Also, pK of protonation of Asp and Glu residues is higher (pK ~ 6–7) near the surface of the hydrophobic membrane compared to bulk aqueous solution, where pK ~ 3–4^10^,^11^. The most effective pH-sensitive tumor targeting agents should sense pH at the surface of cancer cells, where it is the lowest^12^. There are a number of approaches under development for delivery of imaging and therapeutic agents to diseased tissue in a pH-dependent manner. They are based on the use of pH-sensitive polymers, liposomes, nanoparticles and small molecules^13^,^14^,^15^,^16^,^17^,^18^,^19^. Among peptides, family of pHLIP^®^ peptides, linear peptides of 25–35 residues, which insert into cellular membrane and form transmembrane helices are used for targeting of acidic tumors of various origins and other acidic diseased tissues^20^. Application of cyclic peptides in biological sciences has become a subject of major interest because of their enhanced enzymatic stability versus linear peptides^21^. Recently we reported the design and synthesis of homochiral L-cyclic peptides containing arginine (R), tryptophan (W) residues and their application for the nuclear targeting delivery of anti-HIV drugs, phosphopeptides, anticancer drugs, and siRNA^22^,^23^,^24^. These peptides offered several advantages including nuclear delivery of doxorubicin, low cytotoxicity, biocompatibility, hydrophobic drug entrapment through non-covalent interactions, and drug delivery through conjugation. Herein, we designed and introduced for the first time the pH-sensitive negatively charged cyclic peptides and studied their interactions with the lipid bilayer of liposomal and cellular membranes *in vitro* and *in vivo*.

## Results

**Figure 1:**
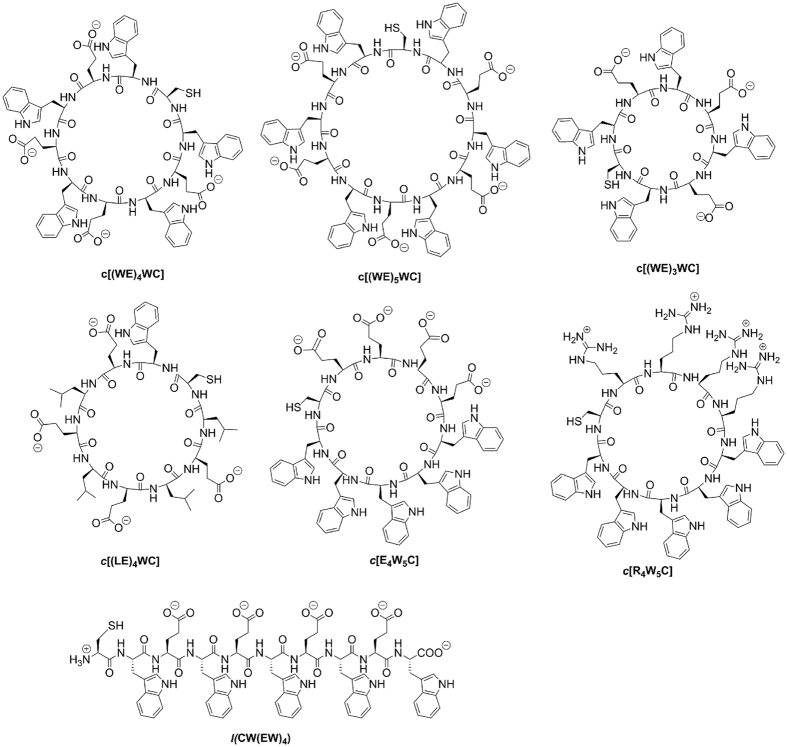
Chemical structures of six cyclic and one linear peptides containing tryptophan (Trp, W), leucine (Leu, L), glutamic acid (Glu, E), arginine (Arg, R) and cysteine (Cys, C) residues.

Among the investigated peptides were one linear and six cyclic peptides (Fig. 1 and Table S1, Supplementary Information). All peptides contained: i) single cysteine (Cys, C) residue for conjugation purposes, ii) at least one tryptophan (Trp, W) for ability to record fluorescence signal, iii) 3–5 protonatable glutamic acid (Glu, E) residues to trigger pH-dependent interaction with the membrane. Three peptides, *c*[(WE)_3_WC], *c*[(WE)_4_WC] and *c*[(WE)_5_WC] had 3, 4, and 5 repeating units of WE, respectively, where W and E were alternating in the cyclic of the peptide. Another peptide, *c*[(LE)_4_WC], had leucine (Leu, L) instead of Trp. The main goal was to investigate the role of aromatic Trp residues in peptide’s interaction with the membrane. Fifth peptide, *c*[E_4_W_5_C], was an asymmetric; it had five Trp residues located on one side of the cycle, while four Glu residues were located on the other side of the cycle. The positively charged cyclic peptide control, *c*[R_4_W_5_C], was also investigated, where Glu residues were replaced by positively charged arginine (Arg, R) residues. Finally, we synthesized one linear *l*(CW(EW)_4_) 10-residue peptide for the comparison with the cyclic peptides. The peptides were synthesized by employing Fmoc/*t*Bu-based solid phase chemistry. As representative examples, the synthesis of *l*(CW(EW)_4_ and *c*[(WE)_4_CW] peptides are depicted in the Scheme (Scheme S1, Supplementary Information). All peptides were purified (95–99%) by reverse phase HPLC.

Fluorescence and CD spectroscopies were employed to monitor pH-dependent peptide’s interaction with the lipid bilayer of liposomes (Figures S1 and S2, Table S2). Biophysical studies were carried out at pH 8 and pH 3 to ensure completeness of the transitions, and to perform measurements in the states of thermodynamic equilibrium. All peptides demonstrated pH-dependent partitioning into the membrane. Asymmetric cyclic peptide with Trp residues located on one side of the cycle, *c*[E_4_W_5_C], most probably partitioned into the membrane facing Trp residues into the bilayer and exposing charged Glu residues to the extracellular space. The drop of pH led to the protonation of carboxyl groups of Glu residues, which increased peptides hydrophobicity and promoted further partitioning of the peptides into the bilayer. As a result, positions of maximum of fluorescence spectra shifted to 6–9 nm to short wavelengths, which is indicative of changes of microenvironment of Trp residues from polar to hydrophobic^25^,^26^ (Figure S1 and Table S2). *c*[(WE)_3_WC], *c*[(WE)_4_WC] and *c*[(WE)_5_WC] peptides showed similar CD signals at pH 8, which were altered by interaction with lipid bilayer and drop of pH. The CD signal of *c*[E_4_W_5_C] peptide was different but was also pH-dependent. We did not observe characteristic CD signal of exciton (delocalized, shared electron density) with a characteristic minimum at 232–235 nm^27^. Such an exciton might be formed only in a result of stacking of aromatic amino acids due to the cyclic peptide’s aggregation to form tubular structures. Thus, we concluded that at the concentrations of the peptides used in this study, the formation of tubular structures is unlikely.

**Figure 2:**
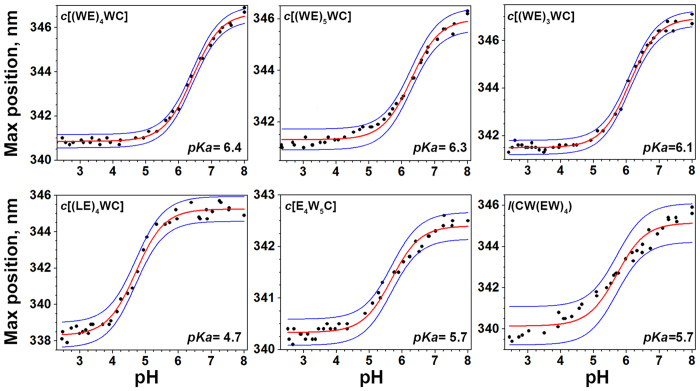
The changes of tryptophan fluorescence are used to follow the partition of the peptides into POPC liposomes as a function of pH. Fitting curves (red lines) and 95% confidence interval (blue lines) are shown.

By monitoring shift of the position of the maximum of fluorescence spectra for the peptides in the result of the pH drop, we established apparent pK of peptide’s partitioning into the bilayer. The pK for most cyclic and linear peptides varied in the range of 5.7–6.4, while the smallest pK value was observed for the cyclic Leu-containing peptide, *c*[(LE)_4_WC] (Fig. 2).

**Figure 3:**
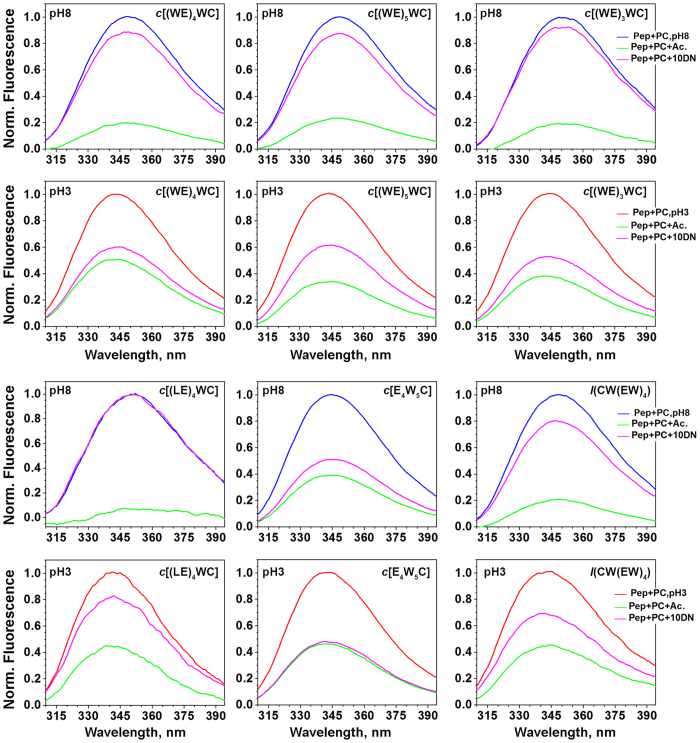
Quenching of fluorescence of peptides in the presence of POPC liposomes at pH 8 (blue lines) or at pH 3 (red lines) by acrylamide (green lines), and 10-DN (magenta lines) are shown. The percentage of quenching is given in Table S3.

**Figure 4:**
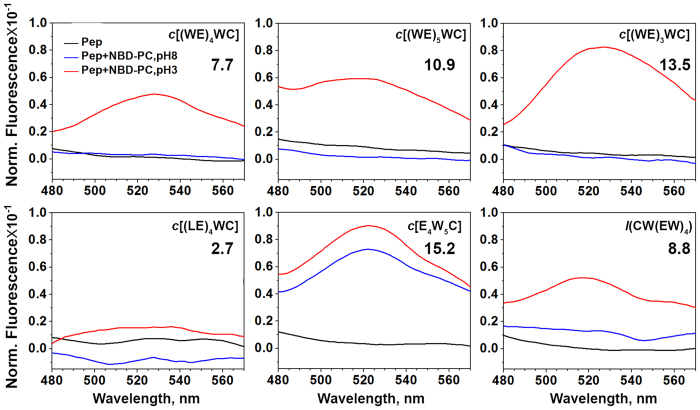
NBD fluorescence spectra of peptides in phosphate buffer at pH 8 (black lines) and in presence of asymmetrically labelled POPC liposomes containing NBD at the inner leaflet at pH 8 (blue lines) and at pH 3 (red lines) are shown. The numbers indicate an increase of FRET at pH 3 compared to the peptide fluorescence in phosphate buffer at pH 8.

To establish localization of the peptides within a lipid bilayer of membrane, dual quenching assay^28^ was employed (Fig. 3 and Table S3). Effective quenching of fluorescence by acrylamide would occur only for tryptophan residues exposed to polar parts of the outer or inner leaflets of the bilayer. At the same time, tryptophan residues located in the middle of a membrane would be effectively quenched by 10-DN. The result of the dual quenching assay allows establishing if tryptophan residues were located in the middle of a membrane or close to the polar headgroups of the bilayer. However, it does not allow distinguishing between locations at the outer or inner leaflets of the bilayer. Therefore, we also performed Förster resonance energy transfer (FRET) assay^29^ (Fig. 4). First, symmetrically-labelled by NBD dye, POPC liposomes were prepared. Then, membrane-impermeable dithionite was used to chemically modify exposed NBD, which led to the quenching of NBD fluorescence. Thus, NBD dyes located at the outer leaflet of the bilayer were deactivated and excess of dithionite was removed by gel filtration. As a result, asymmetrically-labelled liposomes with active NBD at the inner leaflet were obtained. FRET was monitored from tryptophan residues of the peptides to NBD. Energy transfer might occur only when both fluorophores are in proximity to each other (within 5–15 Å). Thus, when tryptophan residues located at the outer leaflet of the bilayer no significant energy transfer to NBD at the inner leaflet would occur (the distance is about 50–60 Å). The *c*[E_4_W_5_C] demonstrated the highest quenching by 10-DN and the highest FRET at pH 8 indicating the internal position of Trp residues within the bilayer of a membrane. All other peptides were located at the outer leaflet of the bilayer at pH 8. Drop of pH promoted partitioning of all cyclic peptides into bilayer, which highest FRET for the *c*[E_4_W_5_C] at pH 3. Among *c*[(WE)_3_WC], *c*[(WE)_4_WC] and *c*[(WE)_5_WC] peptides, the peptide with the smallest cycle, *c*[(WE)_3_WC], showed the deeper partitioning into the membrane. Linear peptide, *l*(CW(EW)_4_), also demonstrated some partitioning into bilayer in the result of the pH drop.

**Figure 5:**
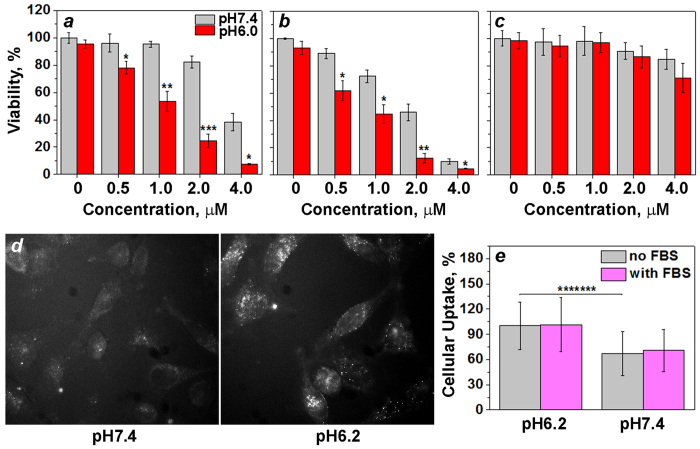
(**a**–**c**) Concentration- and pH-dependent inhibition of HeLa cells proliferation was monitored at 48 hours after incubation of cells within (**a**) cleavable *c*[(WE)_4_WC]-S-S-amanitin, (**b**) cleavable *c*[E_4_W_5_C]-S-S-amanitin and (**c**) non-cleavable *c*[E_4_W_5_C]-amanitin constructs for 3 hours at normal (pH 7.4) and low (pH 6.0) pHs followed by constructs removal and keeping cells in DMEM with 10% FBS at pH 7.4. (**d**) HeLa cells were treated with FITC-labelled *c*[E_4_W_5_C] peptide conjugate (5 μM) for 30 min at pH 7.4 or 6.2, followed by washing at pH 7.4 in both cases, addition of Trypan Blue for 5 min and live cell imaging. (**e**) Cellular uptake of Alexa546-labelled *c*[E_4_W_5_C] peptide conjugates (5 μM) treated with HeLa cells for 6 hours in L-15 media at pH 6.2 in absence and presence of 4% of FBS, followed by washing and counting of fluorescent signal from cells using cellometer. Data are presented as mean ± St.D. The two-tailed unpaired Student’s t-test was used to calculate p-levels.

Since all peptides exhibited pH-dependent interactions with the lipid bilayer of membrane and no cytotoxicity was observed (Figure S3), we proceeded to the experiments on cultured cancer cells. Symmetric, *c*[(WE)_4_WC], and asymmetric, *c*[E_4_W_5_C], cyclic peptides were evaluated for their ability to move polar cargo across the membrane. The experiments on live cells were performed at physiologically relevant pHs, such as pH 7.4 (extracellular pH of normal cells) and pH 6.0 (extracellular pH in the vicinity of cancer cells^12^). As a polar cargo, we used amanitin, which is a cell-impermeable cyclic peptide of molecular mass similar to the masses of investigated pH-sensitive cyclic peptides. Amanitin is a deadly toxin, which inhibits RNA polymerase II if transferred across the lipid bilayer of the plasma membrane^30^. The pH- and concentration-dependent cell death was observed after treatment of HeLa cells for just 3 hours with *c*[(WE)_4_WC]-S-S-amanitin (*c*[(WE)_4_WC]-SPDP-amanitin) and *c*[E_4_W_5_C]-S-S-amanitin (*c*[E_4_W_5_C]-SPDP-amanitin) (Fig. 5a,b). The calculated IC_50_ values for symmetric cyclic peptide amanitin construct, *c*[(WE)_4_WC]-S-S-amanitin, at pH 7.4 and 6.0 were 3.39 ± 0.12 μM and 1.06 ± 0.02 μM, respectively. The calculated IC_50_ values for asymmetric cyclic peptide amanitin construct, *c*[E_4_W_5_C]-S-S-amanitin, at pH 7.4 and 6.0 were 1.68 ± 0.13 μM and 0.74 ± 0.08 μM, respectively. Previously we showed that amanitin alone does not induce cell death at the concentrations used in this study and for the duration of treatment of 2–4 hours^31^. We also tested construct, where amanitin was conjugated to the asymmetric *c*[E_4_W_5_C] cyclic peptide via non-cleavable bond (*c*[E_4_W_5_C]-GMBS-amanitin) (Fig. 5c). The cytotoxic effect for the non-cleavable construct was reduced significantly at both pHs. It might indicate that the peptide-amanitin construct remained within the membrane and cleavage of amanitin from the peptide was required to allow amanitin to reach RNA polymerase II in the nucleus. Alternatively, if peptide-amanitin was translocated into the cytoplasm, the cleavage of amanitin might be required, since affinity of the peptide-amanitin to the RNA polymerase II might be reduced compared to the affinity of free amanitin to the RNA polymerase II.

Based on the obtained results, we proposed that at high/normal pH cyclic peptides were located at the outer leaflet of the bilayer. Triggered by pH drop the protonation of Glu residues enhanced peptide’s hydrophobicity and induced partitioning of the pepetides into the bilayer of the membrane. This assumption was further confirmed by quenching of fluorescence of FITC-labelled asymmetric cyclic peptide by cell impermeable Trypan Blue (Fig. 5d). Trypan Blue is used to quench fluorescence of FITC located in the extracellular space^32^. Cells treated with the FITC-labelled peptide at low pH followed by Trypan Blue quenching showed higher fluorescent signal on cells opposed to the cells treated with the FITC-labelled peptide at normal pH followed by Trypan Blue quenching. It indicates that at normal pH FITC is more exposed to extracellular space (to Trypan Blue) in contrast to low pH treatment. Since FITC fluorescence is a pH-senstive and significantly quenched at low pH (pH 5.0–5.5) in endosomal compartment, we concluded that the FITC-labelled peptide was localazed at the inner leaflet of the plasma membrane rather than in endosomes. Also, the cellular uptake of Alexa546-labelled *c*[E_4_W_5_C] peptide at normal and low pHs in presence and absence of FBS was investigated (Fig. 5e). We observed statistically significant difference in cellular uptake of peptide at normal and low pHs, and no reduction of the uptake in the presence of FBS.

**Figure 6:**
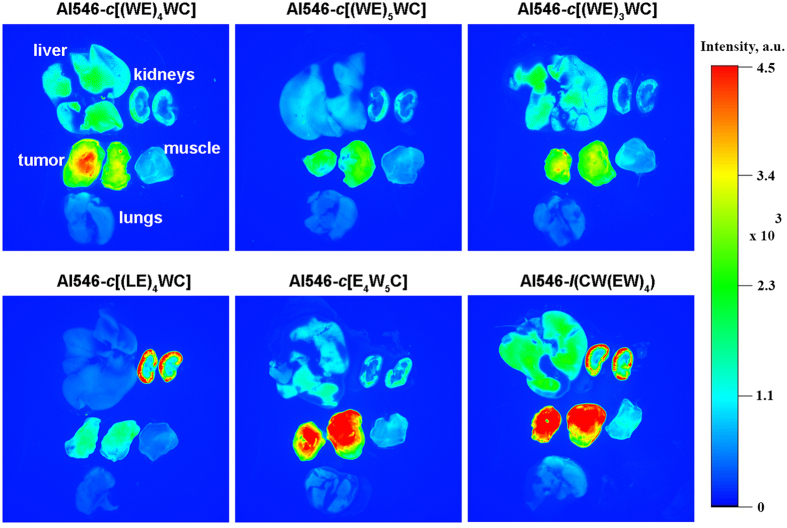
*Ex vivo* fluorescence imaging of tumor, muscle, lungs, liver and kidneys collected at 4 hours after intravenous administration of Alexa546-peptides are shown. Three mice per peptide were used in the study.

Next we proceeded to animal studies to identify the lead peptide demonstrating best tumor targeting. The murine 4T1 xenograft model, which closely mimics stage IV of human breast cancer^33^,^34^, was used in our study. Small 4T1 tumor (tumor volume <150 mm^3^) generates a significant level of lactate and serves as a good model of an aggressive, acidic tumor^35^. All peptides were covalently conjugated with Alexa546-malemide. The fluorescent constructs were given as a single IV injection, and at 4 hours after administration, animals were euthanized followed by necropsy. The mean fluorescence of tumor, muscle, kidney, liver and lungs were recorded and analyzed (Fig. 6). The least targeting was observed for Leu-containing peptide, *c*(LE)_4_CW. At the same time, the highest tumor targeting was monitored for linear peptide, *l*(CW(EW)_4_), symmetric, *c*[(WE)_4_WC], and asymmetric, *c*[E_4_W_5_C], cyclic peptides.

To prove pH-dependent tumor targeting of WE cyclic peptides we tested positively-charged asymmetric cyclic peptide, *c*[R_4_W_5_C], where Glu residues were replaced by Arg residues. This WR-peptide interacted with the lipid bilayer of the membrane at high pH (Figure S4) due to the presence of Trp residues, similar to WE-peptide. At the same time, it does not exhibit pH-dependent changes in interaction with the lipid bilayer of membrane, thus it can serve as a pH-insensitive control for pH-sensitive WE-cyclic asymmetric peptide, *c*[E_4_W_5_C].

**Figure 7:**
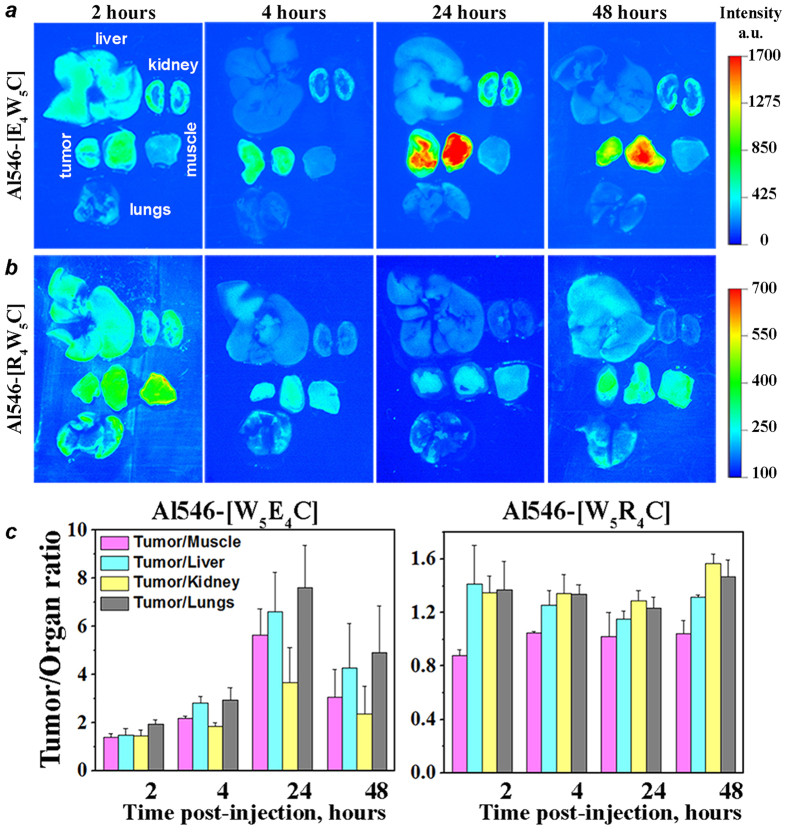
*Ex vivo* fluorescence imaging of tumor, muscle, lungs, liver and kidneys collected at various time points after intravenous administration of Alexa546 asymmetric *c*[E_4_W_5_C] (**a**) and *c*[R_4_W_5_C] (**b**) peptides; (**c**) Tumor/organ ratios calculated from the obtained data. Three mice per time point, per peptide were used in the study.

We performed side-by-side 4T1 tumor targeting and biodistribution investigation of asymmetric pH-sensitive, *c*[E_4_W_5_C], and pH-insensitive, *c*[R_4_W_5_C], peptides. A significant difference in tumor targeting was observed at all time points after IV administration of the constructs (Figs 7a and S5). It resulted in a significant difference between tumor/organ ratios for pH-sensitive and insensitive peptides (Fig. 7b and Table S4). The accumulation of the pH-insensitive peptide in tumor and other organs was very minimal and varied in the range of 0.8 to 1.6 values, which could be attributed to the passive diffusion with blood flow.

## Discussion

**Figure 8:**
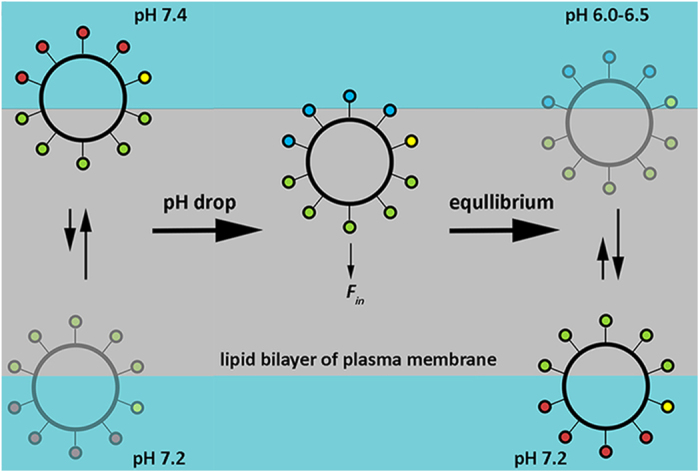
The cyclic peptide molecules distribution between outer and inner leaflets of the lipid bilayer of plasma membrane. At neutral and high pHs, Glu residues are negatively-charged (red circles). Trp residues (green circles) interact with polar headgroups. Cys residue (yellow circle) could be directed into bilayer or away depending on cargo hydrophobicity conjugated with Cys. The majority of cyclic peptides could be found on the outer bilayer of plasma membrane of normal cells compared to the inner bilayer due to the small pH gradient (pHe = 7.4 and pHi = 7.2). A drop of a pH leads to the protonation of Glu residues (blue circles), which enhances peptides hydrophobicity and induces partition into the bilayer.

We have introduced a novel class of cyclic peptides, which demonstrate pH-sensitive interaction with lipid bilayer of membrane of liposomes, cultured cancer cells and tumors in animal model. We tested six cyclic peptides of three different cycle sizes, and different localization of Trp, Glu, Leu and Arg residues within the cycles. The size of investigated peptides is an optimal for the straightforward synthesis. The significantly smaller or larger sized cycles are more challenging to synthesize. The interaction of cyclic peptides with lipid bilayer of membrane has different mechanism compared to the action of linear pH-sensitive peptides such as pHLIP^®^ peptides. Negatively charged Glu residues of cyclic peptides, especially asymmetric one, which showed the best pH-dependent performance, are exposed to the aqueous solution at normal pH. At the same time, indole rings of Trp residues most probably interact with the lipid headgroups (Figs 8 and S6). It was demonstrated previously that aromatic Trp residues have high affinity to lipid headgroups^11^,^36^,^37^,^38^,^39^. Our biophysical titration data indicate that *c*[E_4_W_5_C] cyclic peptide partitions into the lipid bilayer at pH 8. About 7.3 kcal/mol of free energy is released during the process (Figure S7), which indicates on strong interactions of the peptide with the membrane. The high affinity of the peptides to the cellular membranes at normal pH might explain long time of circulation in mice. When pH is lowered, Glu residues are protonated. The pK of Glu residue protonation in the vicinity of the hydrophobic membrane is higher compared to the pK in a solution. Protonation leads to the increase of hydrophobicity of the peptide, which promotes partitioning of the peptide into the bilayer. As a result, about 0.7 kcal/mol of additional free energy is released at low pH (Figure S7). This energy could be used to move cell-impermeable cargo, such as amanitin, across membrane and target acidic tumors. Because Trp residues have higher affinity to the headgroups region compared to the central hydrophobic part of the bilayer, the peptide is equilibrated in the regions of headgroups between inner and outer leaflets of the bilayer. We and others showed that pH equilibrates fast inside a liposome^40^,^41^. Thus, an equal amount of the peptide molecules is distributed between both leaflets of the liposomal membrane with low pH outside and inside of it (Figure S6). However, in the case of live cells, pH inside a cell is normal (7.2–7.4), while bulk extracellular pH is slightly lower (6.5–6.8)^42^,^43^,^44^. However, the pH is at its lowest at the surfaces of cells^9^ and increases with distance from the cellular membrane, becoming normal in the vicinity of blood vessels^45^. Recently, we were able to measure *in vitro* and *in vivo* pH in the vicinity of plasma membrane of cancer cells, the pH values drops to 6.0–6.5 at the surface of cancer cells^12^. The most aggressive cancer cells are the most acidic. Thus, peptides reaching inner leaflet of the bilayer could expose their Glu residues to the cytoplasm, where they are expected to be de-protonated and became charged again. It would reduce the rate of the peptide diffusion back into the membrane and should lead to the shift of the equilibrium toward the accumulation of the peptides at inner leaflet of bilayer of the plasma membrane of cells (Fig. 8).

Thus, cyclic peptides could be considered as a weak acid with multiple protonatable groups, which can diffuse across the bilayer. For weak acids, the intracellular-extracellular distribution, *C*_*i*_/*C*_*e*_, should be calculated according to the following equation:


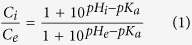


where *pH*_*i*_ and *pH*_*e*_ are the intracellular and extracellular pH values, respectively. Since the cyclic peptides have affinity to the membrane, we consider *C*_*i*_ and *C*_*e*_ as the concentrations of the peptide on inner and outer leaflets, respectively. We established the pKa of membrane partition for the asymmetric cyclic peptide, which equals to 5.7. The calculation shows that at pHe = 7.4 and pHi = 7.2 the concentration ratio at the inner and outer leaflets for asymmetric cyclic peptides is 0.6. However, the same ratio increases to 4.5, 7.8, and 10.9 if extracellular pHe would be 6.5, 6.2 and 6.0, respectively. We assume that the symmetrical WE peptides have the same mechanism of action as an asymmetric peptide, however they have less favorable localization of Trp and Glu residues, which reduces their ability to accumulate at the inner leaflet of bilayer of cellular membranes and target acidic tumors. Leu-containing peptides are less advantageous due to their reduced affinity to the headgroup part of the bilayer and high affinity to the center of the membrane.

A novel class of pH-sensitive cyclic peptides containing tryptophan and glutamic acid residues have potential applications for targeting tumors and translocation of polar cargo molecules across cellular membrane. These peptides might have application not only in targeting of acidic diseased tissue, they might find very interesting applications in cosmetics. It is well known that the natural skin surface pH is on average below 5^46^. Thus, topical application of short (8–10 residues long), very stable cyclic peptides might open an opportunity to tether various cosmetic and skin care products to the skin surface.

## Methods

### Materials

The materials including Fmoc-L-amino acid building blocks, preloaded amino acids on 2-chlorotrityl resin as solid support, and 2-(1*H*−benzotriazol-1-yl)-1,1,3,3-tetramethyluronium hexafluorophosphate (HBTU) used for coupling reagents were purchased from Chem-Impex Int’l Inc., Wood Dale, IL. Piperidine and *N*-methylmorpholine were purchased from Sigma-Aldrich Chemical Co. (Milwaukee, WI). The other chemicals such as *N*,*N*-diisopropylethylamine (DIPEA), cleavage cocktail reagents trifluoroacetic acid (TFA), 1-hydroxy-7-azabenzotriazole (HOAt), *N,N*′-diisopropylcarbodiimide (DIC), acetic acid (AcOH), 2,2,2-trifluoroethanol (TFE), anisole, thioanisole, ethanedithiol (EDT), and anhydrous solvents such as *N*,*N*-dimethylformamide (DMF), dichloromethane (DCM), hexane, acetic acid (AcOH), and 2,2,2-trifluoroethanol (TFE) were purchased from Fisher Scientific, Pittsburg, PA.

### Methodology of Peptide Synthesis

In brief, the linear protected peptide was first assembled. Direct cleavage of peptide-attached resin in the presence of AcOH/TFE/DCM (1:2:7 v/v/v) generated the linear peptide. Cyclization in dilute condition using DIC/HOAt in DMF/DCM solution for 12 hours, followed by the deprotection of the side chain by using cleavage cocktail (TFA:thioanisole:anisole:EDT (90:5:2:3 v/v/v/v) afforded the cyclic peptide. The peptides were synthesized by employing the *N*-(9-fluorenyl)methoxycarbonyl (Fmoc)-solid phase chemistry using PS3 automated peptide synthesizer (Rainin Instrument Co., Inc.) at room temperature. The peptide sequence was assembled on preloaded amino acid on 2-chlorotriyl resin using coupling, activating, and deprotecting reagents using HBTU, *N*-methylmorpholine (0.4 M), and piperidine in DMF (20% v/v), respectively. The amino acids in the peptide sequence were coupled using coupling reagents and activating reagent in DMF for 1 hour followed by washing with DMF 3 times. The deprotection was carried using piperidine (20%, v/v) in DMF for 2 times, 10 minute for each time, followed by washing with DMF (3 times). The appropriate sequence of linear protected peptide was assembled using the synthesizer. *N-* to *C*-terminal cyclization of peptide were achieved by cleavage of protected peptidyl resin by stirring the peptidyl resin in freshly prepared cleavage cocktail of AcOH/TFE/DCM (1:2:7, v/v/v) for 1 hour at room temperature followed by washing the resin with TFE:DCM (2:8 v/v, 2 times). The collected filtrate was evaporated using a rotary evaporator followed by azeotropic removal of acetic acid by addition of hexane and dichloromethane to afford high viscous liquid or solid-protected linear peptide. The crude linear protected peptide was dissolved in excess of solvents DMF:DCM (4:1 v/v) followed by the addition of HOAt/DIC (1:1.1 equiv) for cyclization for 12–48 hours confirmed by MALDI TOF-TOF mass spectrometry. The solvent was evaporated under high reduced pressure in a rotatory evaporator at 40–45 °C to remove DMF. The final cleavage of side chain protection from the peptide were carried out after confirming the peptide cyclization by MALDI mass spectrometer data by shaking the cyclized peptide mixture in cleavage cocktail reagent R (TFA/thioanisole/anisole/EDT (90:5:2:3 v/v/v/v, 10–15 mL) for 2–4 hours followed by precipitation of peptide using cold ether, centrifugation at 2500 rpm and washing with excess of cold ether at 25 °C for 5 min. The crude peptide was purified with semi preparative reversed phase high performance liquid chromatography (RP-HPLC) by using Hitachi L-2455 on a C18 Phenomenex Prodigy reversed-phase column (10 μm, 250 cm × 21.2 cm). The pure peptide was eluted at 15.0 mL/min using a gradient of binary solvent system using water and acetonitrile with 0.1% TFA for 0–100% over 60 min. The pure collected peptide fractions were pooled and lyophilized to provide solid powder in purity of ≥98%. All peptides were characterized by using high resolution time of flight AXIMA-performance MALDI TOF-TOF mass spectrometer (Shimadzu). The above mentioned protocol was applied for the synthesis of all cyclic peptides. The concentration of the peptides was calculated spectrophotometrically by measuring absorbance at 280 nm. The extinction coefficients, ε_280_, M^−1^ cm^−1^, for the peptides are the following: *c*[(WE)_4_WC] = 28,000; *c*[(WE)_5_WC] = 33,600; *c*[(WE)_3_WC] = 2,400; *c*[(LE)_4_WC] = 5,600; *c*[E_4_W_5_C] = 28,000; *l*(CW(EW)_4_) = 28,000.

### Synthesis of Cyclic Peptides

The linear peptide sequence was synthesized on PS3 automated synthesizer as described above in the scale of 0.3 mmol. H-Trp(Boc)-2-chlorotrityl resin (384.6 mg, 0.3 mmol, 0.78 mmol/g) was swelled in DMF, followed by coupling and deprotection cycles to assemble respective amino acids on the peptidyl resin using respective amino acids, such as Fmoc-Glu(OtBu)-OH (382.9 mg, 0.9 mmol), Fmoc-Trp(Boc)-OH (473.9 mg, 0.9 mmol), Fmoc-Cys(Trt)-OH (527.1 mg, 0.9 mmol), and HBTU (341 mg, 0.9 mmol) as the coupling reagent. Fmoc group of *N*-terminal in the peptidyl resin was removed using deprotection cycle, and the resin was transferred to 100 mL round bottom flask. The linear protected peptide was cleaved by shaking peptidyl resin in cleavage cocktail AcOH/TFE/DCM (1:2:7 v/v/v, 50 mL) for 1 hour followed by washing the resin using TFE:DCM (2:8 v/v, 10 mL, 2 times). The combined filtrate was evaporated to dryness with the subsequently addition of hexane (50 mL × 3) and DCM (10 mL × 3) to remove acetic acid, which provided solid white crude protected peptide ready for cyclization. The cyclization was carried out by dissolving the solid peptide in anhydrous DMF/DCM (250 mL, 4:1 v/v) under nitrogen using DIC (155.0 μL, 0.99 mmol) and HOAt (122.5 mg, 0.9 mmol) with stirring at room temperature for 24 hours. The cyclized product was confirmed by taking a small aliquot of the reaction mixture and cleavage with reagent R and using MALDI. After cyclization was confirmed, the solvents were evaporated under high reduced pressure, and the side chain protections were removed by addition of cleavage cocktail of reagent R, TFA/thioanisole/anisole/EDT (15 mL, 90:5:2:3 v/v/v/v), and shaking at room temperature for 3 hours. The peptides were precipitated, centrifuged, and washed with cold diethyl ether to yield the crude white solid peptide. The peptides were dissolved in H_2_O/CH_3_CN with 0.1% TFA and purified using RP HPLC. Then, the pure fractions were collected, concentrated and lyophilized to afford pure solid white powder of *c*[(WE)_4_WC] peptide. MALDI-TOF (m/z) [C_78_H_83_N_15_O_18_S]: calcd, 1549.6; found, 1572.3 [M + Na ]^+^; *c*[(WE)_5_WC]: MALDI-TOF (m/z) [C_94_H_100_N_18_O_22_S]: calcd, 1864.7; found, 1865.4 [M + H]^+^; *c*[(WE)_3_WC]: MALDI-TOF (m/z) [C_62_H_66_N_12_O_14_S]: calcd, 1234.5; found, 1234.7 [M]^+^; *c*[(LE)_4_WC]: MALDI-TOF (m/z) [C_58_H_87_N_11_O_18_S]: calcd, 1257.6; found, 1258.2 [M + H]^+^; *c*[E_4_W_5_C]: MALDI-TOF (m/z) [C_78_H_83_N_15_O_18_S]: calcd, 1549.6; found, 1549.1 [M]^+^. A similar procedure was used for the synthesis of *c*[R_4_W_5_C] except using Fmoc-Arg(Pbf)-OH instead of Fmoc-Glu(OtBu)-OH. *c*[R_4_W_5_C]: MALDI-TOF (m/z) [C_82_H_103_N_27_O_10_S]: calcd, 1657.8; found, 1658.5 [M + H]^+^.

### Synthesis of *l*(CW(EW)_4_)

The linear peptide was assembled as described above using H-Trp(Boc)-2-chlorotrityl resin (384.6 mg, 0.3 mmol 0.78 mmol/g) in reaction vessel. The peptide sequence was assembled using the appropriate amino acid building blocks Fmoc-Glu(OtBu)-OH (382.9 mg, 0.9 mmol), Fmoc-Trp(Boc)-OH (473.9 mg, 0.9 mmol), Fmoc-Cys(Trt)-OH (527.1 mg, 0.9 mmol), and HBTU (0.9 mmol, 341 mg) as the coupling reagent. The final *N*-terminal Fmoc group was deprotected. The peptide was cleaved from the resin and side chain was deprotected by reaction of the peptidyl resin with freshly prepared cleavage cocktail reagent R, TFA/thioanisole/anisole/EDT (15 mL, 90:5:2:3 v/v/v/v), for 3 hours at room temperature. The linear protected peptide was precipitated, centrifuged, and purified by using RP-HPLC as mentioned above to yield *l*(CW(EW)_4_). MALDI-TOF (m/z) [C_78_H_85_N_15_O_19_S]: calcd, 1567.6; found, 1606.1 [M + K]^+^.

### Labeling of Peptides with Fluorescent Dyes

Peptides were conjugated with Alexa546- and Fluorescein-5-maleimide (Life Technologies) in DMF at a ratio of 1.2:1 and incubated at room temperature for about 6 hours and then at 4 °C until the conjugation reaction was completed. 50 mM of sodium phosphate/150 mM NaCl buffer pH 7.0 (saturated with argon) was added to the reaction mixture (1/10 of the total volume). The reaction progress was monitored by the reverse phase HPLC. The products were purified by the reverse phase HPLC, lyophilized and characterized by SELDI-TOF mass spectrometry. The concentration of the constructs was determined by absorbance at 556 and 494 nm using molar extinction coefficients of 93,000 M^−1^·cm^−1^ for Alexa546 and 68,000 M^−1^·cm^−1^ for Fluorescein-5 (FITC).

### Synthesis of Peptide-Amanitin Constructs

We conjugated symmetric *c*[(WE)_4_WC] and asymmetric *c*[E_4_W_5_C] peptides with alpha-amanitin (Sigma-Aldrich) via cleavable S-S bond. Furthermore, asymmetric *c*[E_4_W_5_C] peptide was labelled with amanitin via non-cleavable bond. The conjugation scheme consisted of 2 steps: i) OH group of amanitin was conjugated with NHS group of the cleavable crosslinker, SPDP, *N*-succinimidyl 3-(2-pyridyl-dithio)-propionate or the non-cleavable crosslinker, GMBS, *N*-γ-maleimidobutyryl-oxysuccinimide ester (both crosslinkers were from (Thermo Scientific) in 50 mM sodium phosphate/150 mM NaCl buffer pH 8.0 at a ratio 1:20 at room temperature for 4 hours to get SPDP-amanitin or GMBS-amanitin. The products were purified by the reverse phase HPLC on Zorbax SB-C18 column (9.4 × 250 mm, 5-Micron). SPDP-amanitin was eluted using a gradient: 0–25%, 40 min (water and acetonitrile with 0.05% TFA) and lyophilized. *c*[(WE)_4_WC] and *c*[E_4_W_5_C] peptides were incubated with SPDP-amanitin or GMBS-amanitin in 100 mM sodium phosphate/150 mM NaCl buffer pH 7.8 (saturated with argon) at a ratio 1:1 at room temperature for 1 hour to obtain amanitin-SPDP-peptides or amanitin-GMBS-peptide, respectively. The products were purified by the reverse phase HPLC on Zorbax SB-C18 column (9.4 × 250 mm, 5-Micron) using gradient 10–55%, 40 min (water and acetonitrile with 0.05% TFA). The products were lyophilized and characterized by SELDI-TOF mass-spectrometry. The calculated and obtained masses for the peptides were the following: *c*[E_4_W_5_C]-SPDP-amanitin: SELDI-TOF (m/z) [C_120_H_139_N_25_O_33_S_3_]: calcd, 2553.9; found 2555.5 [M + H]^+^; *c*[E_4_W_5_C]-GMBS-amanitin: SELDI-TOF (m/z) [C_125_H_144_N_26_O_35_S_2_]: calcd, 2632.9; found 2634.6 [M + H]^+^, and 2657.8 [M + Na]^+^; *c*[(WE)_4_WC]-SPDP-amanitin: SELDI-TOF (m/z) [C_120_H_139_N_25_O_33_S_3_]: calcd, 2553.9; found 2554.5 [M + 1]^+^.

### Liposome Preparation

Large unilamellar vesicles (LUVs) were prepared by extrusion. POPC, 1-palmitoyl-2-oleoyl-sn-glycero-3-phosphocholine (Avanti Polar Lipids), or a mixture of POPC with 0.5% of 18:1 NBD-PE, 1,2-dioleoyl-*sn*-glycero-3-phosphoethanolamine-N-7-nitro-2-1,3-benzoxadiazol-4-yl ammonium salt (Avanti Polar Lipids) were dissolved in chloroform, desolvated on a rotary evaporator, and dried under high vacuum for several hours. The phospholipid film was then rehydrated in 10 mM phosphate buffer pH 8.0, vortexed until the lipid bilayer was completely dissolved, and repeatedly (15–21 times) extruded through the membranes with 50 nm pore sizes to obtain LUVs.

### Steady-State Fluorescence and CD

Freshly prepared peptides and POPC vesicles were mixed to have 5 μM of a peptide and 1.25 mM of lipids in the final solution. Steady-state fluorescence measurements were carried out on a PC1 spectrofluorometer (ISS, Inc.) under temperature control at 25 °C. Tryptophan fluorescence was excited at 280 nm (there is no Phe or Tyr in the peptides) and recorded with the excitation and emission slits set at 1 nm. The polarizers in the excitation and emission paths were set at the “magic” angle (54.7° from the vertical orientation) and vertically (0°), respectively. Steady state CD measurements were carried out in MOS 450 spectropolarimeter (Bio-Logic, Inc.) with the same concentrations of peptide and lipids used in fluorescence measurements.

### pH-Dependence

pH-dependent partitioning of the peptides into a lipid bilayer of the membrane was investigated by the shift of the position of the fluorescence spectral maximum for the peptides in the presence POPC liposomes induced by a drop of pH from 8 to 2.5 by the addition of HCl. The peptides were incubated overnight with 50-nm POPC liposomes (final concentration of the peptides and POPC in solution was 5 μM and 1 mM, respectively), and pH decrease was achieved by the addition of aliquots of 4, 2, 1 and 0.1 M HCl. pH was measured by micro-electrode probe (Thermo Electron Corporation, Orion Ross Micro pH electrode). Fluorescence spectra were recorded at each pH value. The spectra were analyzed by the decomposition algorithms using on-line PFAST toolkit^47^ (Protein Fluorescence and Structural Toolkit: http://pfast.phys.uri.edu/) to establish the position of the emission maximum. Finally, the positions of the fluorescence spectral maxima (*λ*_max_) were plotted versus pH, and the Henderson–Hasselbalch equation was used to fit the data (using Origin 8.5 software):


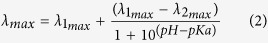


where 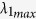 and 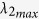 are the beginning and the end of the transition, respectively, and *pK*a – is the midpoint of the transition.

### Titration

Samples containing 5 μM of peptides at pH 8 and pH 3, and varying concentrations of lipids of the 50 nm POPC liposomes were prepared. The fluorescence spectra of peptides in all samples were measured at 280 nm excitation at 25 °C. A series of POPC blanks with the same concentrations of lipids were measured with the same instrument settings and were subtracted from the corresponding fluorescence spectra of peptides in the presence of POPC. The areas under the emission spectra were calculated and the values were normalized to the first point (the emission of the peptide in the absence of POPC). The titration data were fitted by the peptide-membrane partition model to calculate the mole-fraction partition coefficient, *K*


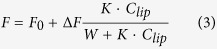


where *F*_*0*_ is the fluorescence intensity at zero concentration of lipids (in our case *F*_*0*_ = 1); Δ*F* if the fluorescence increase in the result of the titration with lipids; *C*_*lipids*_ is the concentration of lipids; *W* is the molar concentration of water (55.3 M). Nonlinear least squares curve fitting procedure using Levenberg-Marquardt algorithm was implemented in Origin 8.5.0 SR1. The Gibbs free energy (Δ*G*) was calculated according to the equation:


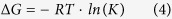


where *R* is the gas constant and *T* is the temperature in Kelvin.

### Dual Quenching

POPC liposomes without and with 10% of the lipids replaced by 10-doxylnonadecane (10-DN) (Avanti Polar Lipids) were prepared in 10 mM citrate-phosphate buffer pH 8.0. Peptides and POPC liposomes were mixed to generate final concentrations of 7 μM peptide and 2.1 mM POPC without and with 10-DN. In some of the samples, the pH was lowered to pH 4 by addition of aliquot of 2 M citric acid, and other samples were kept at pH 8. Quencher, acrylamide (Sigma-Aldrich), was added to the samples of POPC liposomes containing no 10-DN, to have final concentration of 235 mM acrylamide. Concentration of peptides in all samples was kept constant. To observe quenching of tryptophan fluorescence by 10-DN or acrylamide, the tryptophan fluorescence was recorded as described above. The appropriate POPC blanks were measured and subtracted from the measured spectra before analysis. The percentage of quenching was calculated.

### NBD-FRET

First, symmetrically NBD-labelled POPC liposomes containing 0.5% of NBD-PE were prepared. Next, 1.2 mL of 6 mM of symmetrically NBD-labelled POPC liposomes were incubated with 150 μL of 1 M freshly prepared membrane-impermeable dithionite in buffer at pH 8.0 to chemically deactivate of NDB only at the outer leaflet of the bilayer and obtain asymmetrically NBD-labelled POPC liposomes. The decrease of NBD fluorescence occurring in the result of quenching of NBD by dithionite was monitored at the excitation of 463 nm and emission at 530 nm. The dithionite quenching led to the reduction of about 60–65% of NBD fluorescence signal corresponding to the quenching of NBD at the outer leaflet of the bilayer. Next, POPC solution was passed through a G-10 sephadex (Sigma-Aldrich) fast spin column to remove the excess of dithionite. Asymmetrically labelled POPC liposomes were incubated with peptides at concentrations indicated above, and FRET from tryptophan residues to NBD at the inner leaflet of bilayer was monitored at 280 nm excitation wavelength, and emission was recorded from 310 to 580 nm.

### Cell Lines

Human cervix adenocarcinoma (HeLa) cells were acquired from the American Type Culture Collection. Cells were cultured in Dulbecco’s Modified Eagle’s Medium (DMEM) supplemented with 10% fetal bovine serum (FBS), 10 μg/mL of ciprofloxacin in a humidified atmosphere of 5% CO_2_ and 95% air at 37 °C.

### Cytotoxic Assay

HeLa cells were loaded in the wells of 96-well plates (~5,000 cells per well) and incubated overnight. Growth medium was replaced with the medium without FBS pH 6.2 or pH 7.4 containing increasing amounts of constructs (5, 10, 20, and 40 μM). The same volume of DMEM medium supplemented with 20% FBS, pH 7.4 was added after 2 hours of treatment. After 48 hours of incubation a colorimetric reagent (CellTiter 96 AQ_ueous_ One Solution Assay, Promega) was added for 1 hour followed by measuring absorbance at 490 nm to assess cell viability. All samples were prepared in triplicate.

### Proliferation Assay

HeLa cells were loaded in the wells of 96-well plates (~5,000 cells per well) and incubated overnight. Growth medium was replaced with the medium without FBS pH 6.0 or pH 7.4 containing increasing amounts of peptide-amanitin construct (0.5, 1, 2, and 4 μM). The construct was removed from cells after 3 hours. After 48 hours of cell incubation in standard growth medium, a colorimetric reagent (Cell Titer 96 AQ_ueous_ One Solution Assay, Promega) was added for 1 hour followed by measurements of absorbance at 490 nm to assess cell viability. All samples were prepared in triplicate.

### Fluorescent Microscopy

HeLa cells (8,000 cells per dish) were seeded in the center of a 35-mm dish with a 10-mm glass-bottom window coated with collagen (MatTek Corp). Next day cells were incubated with 5 μM of FITC-labelled *c*[E_4_W_5_C] peptide for 30 min in DMEM medium without FBS at pH 7.4 or 6.2. Cells were washed 5 times at pH 7.4 and 0.4% Trypan Blue was added for 5 min (1/10 of the total volume). Fluorescent images were acquired with a Retiga CCD camera (Qimaging) mounted to an inverted Olympus IX71 microscope (Olympus America, Inc.).

### Cellular uptake

HeLa cells (150,000 cells per sample of total volume of 500 μL) were incubated with 5 μM of Alexa546-labelled peptide in Leibovitz’s (L15) medium at pH 6.2 and 7.4 in the presence or absence of 4% of fetal bovine serum (FBS) for 6 hours, followed by extensive washing with L15 medium. The cellular uptake of the constructs was measured by fluorescent signal from cells counted using cellometer (Cellometer Vision CBA, Nexcelom). 4% of FBS was used to mimic the amount of albumin in whole blood, which contains 45% of red blood cells, white blood cells and platelets suspended in plasma (about 55% of volume). Plasma is composed of about 92% of water, 1% of vitamins, sugars, salts, minerals, hormones and 7% of vital proteins including albumin, gamma globulins and other clotting factor. Thus, the amount of albumin in whole blood is expected to be less than 3.6%.

### *Ex Vivo* Fluorescence Imaging

All animal studies were conducted according to the animal protocol AN04-12-011 approved by the Institutional Animal Care and Use Committee at the University of Rhode Island, in compliance with the principles and procedures outlined by NIH for the Care and Use of Animals. 4T1 breast tumors were established by subcutaneous injection of 4T1 cells (8 × 10^5^ cells/0.1 mL/flank) in the right flank of adult female BALB/c mice (about 19–22 g weight) obtained from Harlan Laboratories. When tumors reached about 6 mm in diameter single tail vein injections of 100 μl of 40 μM Alexa546-peptides were performed. Control mice bearing tumor used to establish an auto fluorescence signal did not receive fluorescent peptides. At 4 hours post-injection euthanization and necropsy was performed followed by *ex vivo* imaging of tumor, kidneys, liver, lungs and muscle. Mean fluorescence intensity of tumor and organs was calculated using Kodak software.

### Statistical analysis

Statistically significant differences were determined by two-tailed unpaired Student’s t-test (p-level < 0.05 was taken as significant).

## Additional Information

**How to cite this article**: Weerakkody, D. *et al*. Novel pH-Sensitive Cyclic Peptides. *Sci. Rep.* **6**, 31322; doi: 10.1038/srep31322 (2016).

## Supplementary Material

Supplementary Information
