# Supplementary material for: Novel pH-Sensitive Cyclic Peptides
Source: Sci Rep. 2016 Aug 12;6:31322. doi: 10.1038/srep31322 (PMC4981864; doi:10.1038/srep31322)
Supplement: Supplementary Information [file srep31322-s1.pdf]

Supporting Information

Novel pH-Sensitive Cyclic Peptides

Dhammika Weerakkody[1], Anna Moshnikova[1], Naglaa Salem El-Sayed[2,3,4], Ramona-Cosmina Adochite[1], Gregory Slaybaugh[1], Jovana Golijanin[1], Rakesh K. Tiwari[2,3], Oleg A. Andreev[1], Keykavous Parang[2,3,*], Yana K. Reshetnyak[1,*]

[1]Department of Physics, University of Rhode Island, Kingston, RI 02881, US
[2]Department of Biomedical and Pharmaceutical Sciences, Chapman University School of Pharmacy, 9401 Jeronimo Road, Irvine, CA 92618, US
[3]Department of Biomedical and Pharmaceutical Sciences, College of Pharmacy, University of Rhode Island, 7 Greenhouse Road Kingston, RI 02881, US
[4]Cellulose and Paper Department, National Research Center, Dokki 12622, Cairo, Egypt

[*]reshetnyak@uri.edu

Tables

Table S1. Properties of the synthesized peptides. All peptides were prepared with 95-99% of purity.

Peptide	Calculated M.W.	Found M.W.	Retention time in HPLC
***c*[(WE)$_4$WC]**	1549.5761	1572.2693 $[M + Na]^+$	35.2-36.7
***c*[(WE)$_5$WC]**	1864.6980	1865.3943 $[M + H]^+$	35.8-37.2
***c*[(WE)$_3$WC]**	1234.4542	1234.7385 $[M]^+$	35.0-36.0
***c*[(LE)$_4$WC]**	1257.5951	1258.2421 $[M + H]^+$	35.2-36.3
***c*[E$_4$W$_5$C]**	1549.5761	1549.1430 $[M]^+$	36.6-37.7
***l*(CW(EW)$_4$)**	1567.5867	1606.1204 $[M + K]^+$	32.3-33.2
***c*[R$_4$W$_5$C]**	1657.8102	1658.5018 $[M + H]^+$	32.0-32.1

Table S2. The spectral parameters of the peptides in phosphate buffer at pH 8, in the presence of POPC liposomes at pH 8 and pH 3 are presented. The parameters were obtained from the analysis of the fluorescence spectra shown in Figure S1: the maximum position of the fluorescence spectrum λ_{max}, in nm; ***S*** – the normalized area under the spectra (normalization was done on the area under the spectrum for peptides at pH 8 in absence of POPC liposomes, black lines on Figure S1).

Peptide	λ_{max}, nm			***S***	
	**Pep, pH8**	**Pep-PC, pH8**	**Pep-PC, pH3**	**Pep-PC, pH8**	**Pep-PC, pH3**
***c*[(WE)$_4$WC]**	350.5±0.2	347.5±1.2	341.1±0.9	1.1±0.1	1.6±0.2
***c*[(WE)$_5$WC]**	350.3±0.2	346.7±1.2	341.2±0.4	1.5±0.3	2.1±0.3
***c*[(WE)$_3$WC]**	351.0±0.2	349.1±0.7	341.7±0.2	0.9±0.1	1.7±0.3
***c*[(LE)$_4$WC]**	348.7±0.3	348.2±0.6	339.6±1.2	1.1±0.0	1.1±0.1
***c*[E$_4$W$_5$C]**	350.6±0.2	342.8±0.4	340.2±0.4	2.4±0.3	2.4±0.5
***l*(CW(EW)$_4$)**	353.0±0.7	346.6±0.7	339.8±0.6	2.3±0.4	1.6±0.4

Table S3. The percentage of the peptide's fluorescence quenching by addition of acrylamide (AC) or 10-DN at pH 8 and pH 3 in the presence of POPC liposomes are shown. The values were obtained from the analysis of the fluorescence spectra shown in Figure 4.

	Pep-PC+AC pH8	Pep-PC+ 10-DN pH8	Pep-PC+AC pH3	Pep-PC+ 10-DN pH3
***c*[(WE)$_4$WC]**	82	11	50	40
***c*[(WE)$_5$WC]**	78	13	68	40
***c*[(WE)$_3$WC]**	83	6	63	47
***c*[(LE)$_4$WC]**	96	0	59	17
***c*[E$_4$W$_5$C]**	61	49	54	52
***l*(CW(EW)$_4$)**	81	20	56	31

Table S4. Tumor/Organ ratios shown on Figure 7c.

	Tumor/Muscle	Tumor/Liver	Tumor/Kidney	Tumor/Lung
**Al546-*c*[E$_4$W$_5$C]**				
**2 hours**	1.39±0.14	1.47±0.30	1.45±0.25	1.93±0.19
**4 hours**	2.19±0.10	2.83±0.26	1.86±0.15	2.95±0.51
**24 hours**	5.64±1.11	6.61±1.62	3.66±1.46	7.60±1.77
**48 hours**	3.05±1.17	4.29±1.85	2.37±1.15	4.91±1.94
**Al546-*c*[R$_4$W$_5$C]**				
**2 hours**	0.88±0.04	1.42±0.29	1.35±0.12	1.37±0.21
**4 hours**	1.05±0.01	1.26±0.11	1.34±0.14	1.34±0.07
**24 hours**	1.02±0.18	1.15±0.06	1.29±0.07	1.24±0.08
**48 hours**	1.04±0.10	1.31±0.02	1.56±0.07	1.47±0.12

Schemes

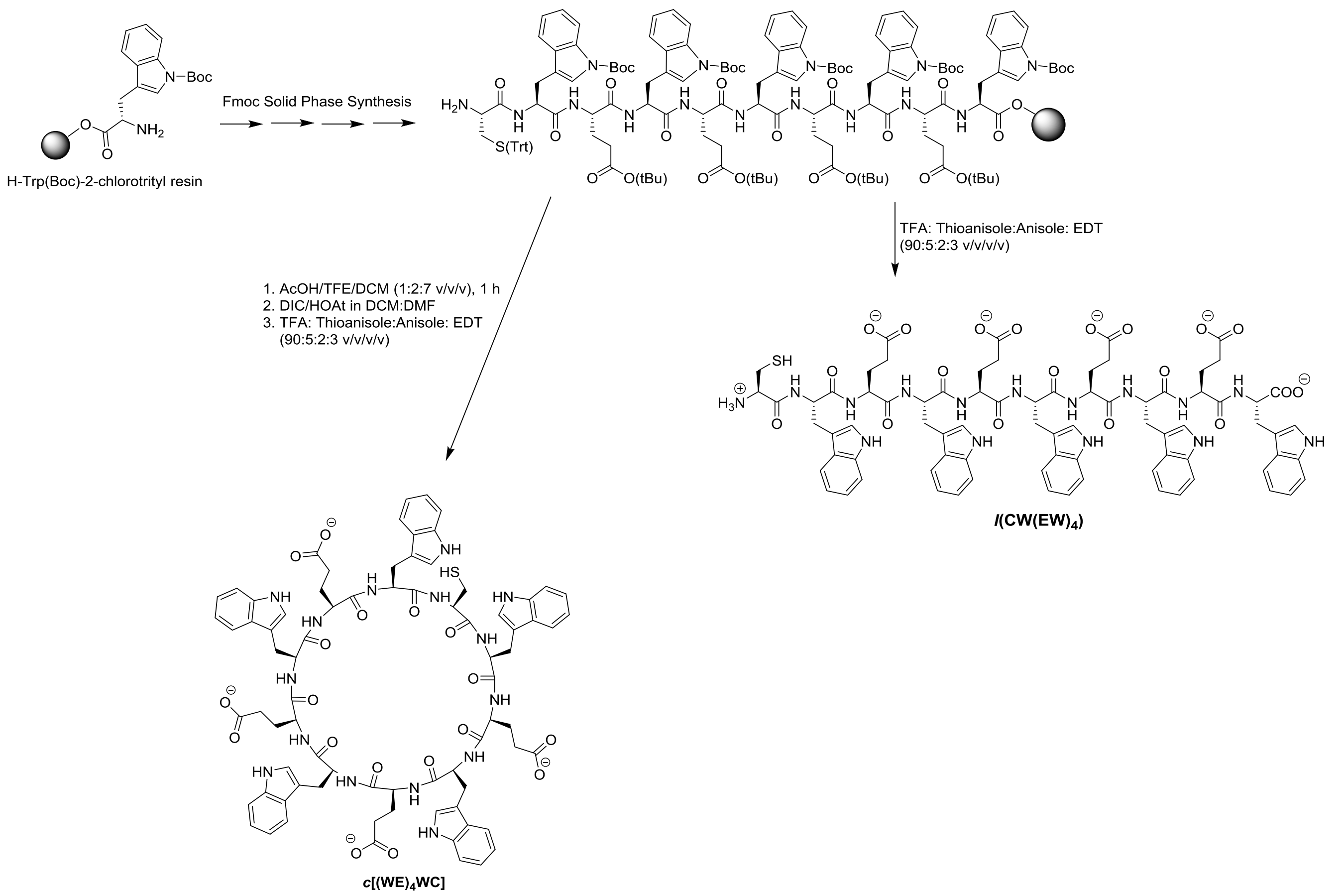

Scheme S1. Synthesis of *l*(CW(EW)$_4$) and *c*[(WE)$_4$WC] peptides.

Figures

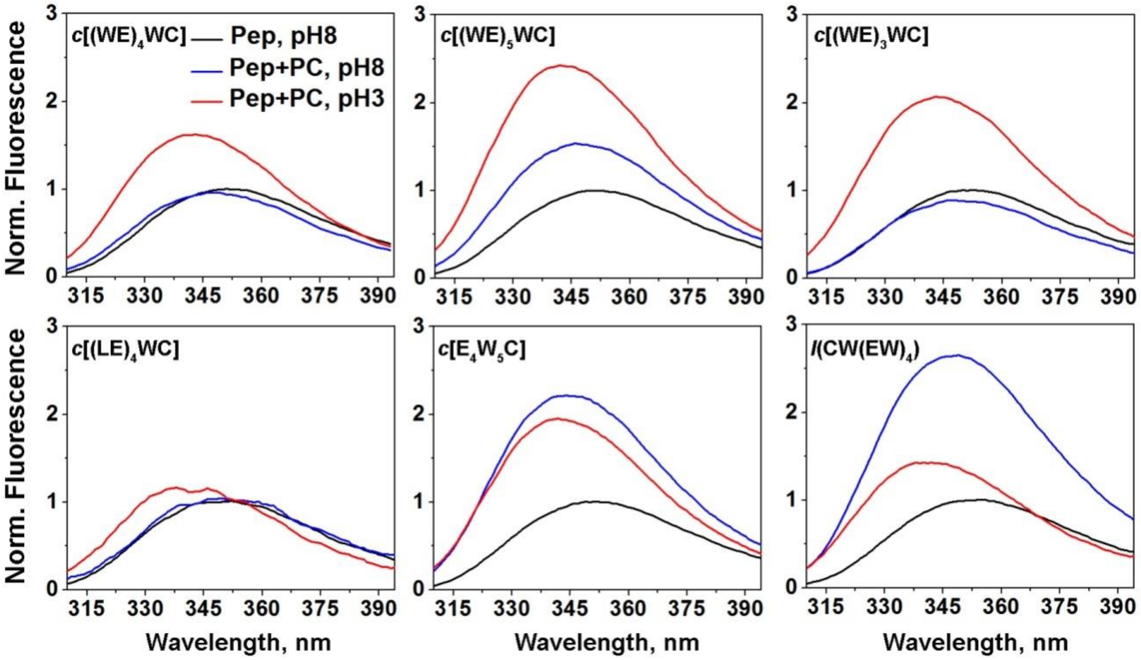

Figure S1. The fluorescence of the peptides in phosphate buffer at pH 8 (black lines) and in the presence of POPC liposomes at pH 8 (blue lines) and pH 3 (red lines) are shown, the excitation wavelength was 280 nm. The spectral parameters are given in Table S2.

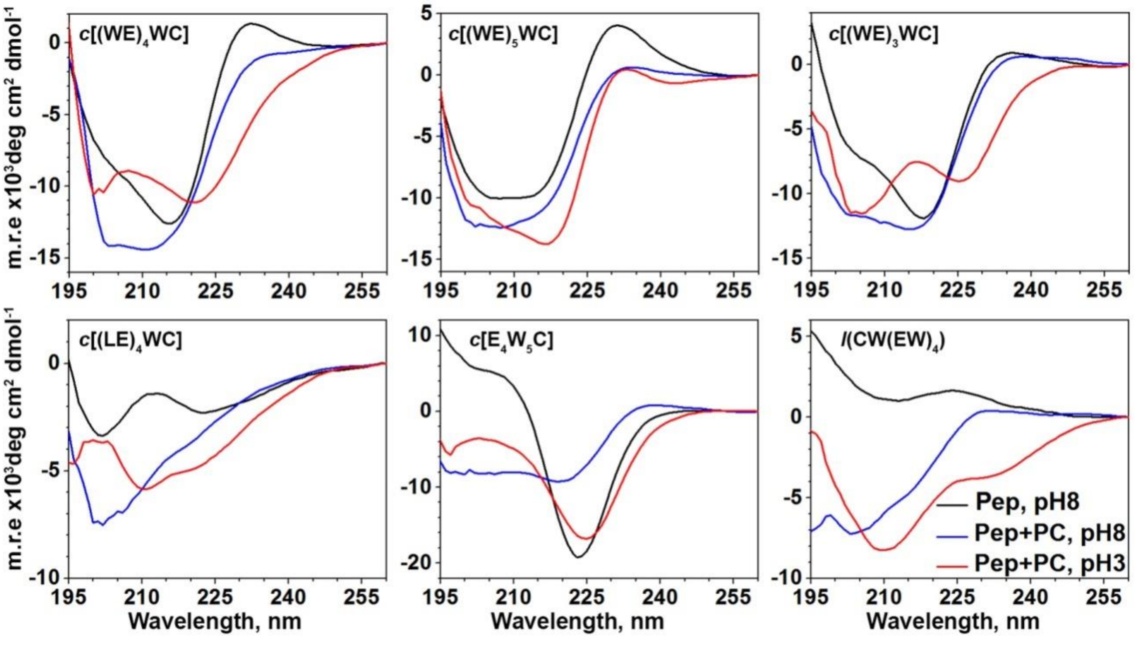

Figure S2. The circular dichroism of the peptides in phosphate buffer at pH 8 (black line) and in presence of POPC liposomes at pH 8 (blue line) and pH 3 (red line) are shown.

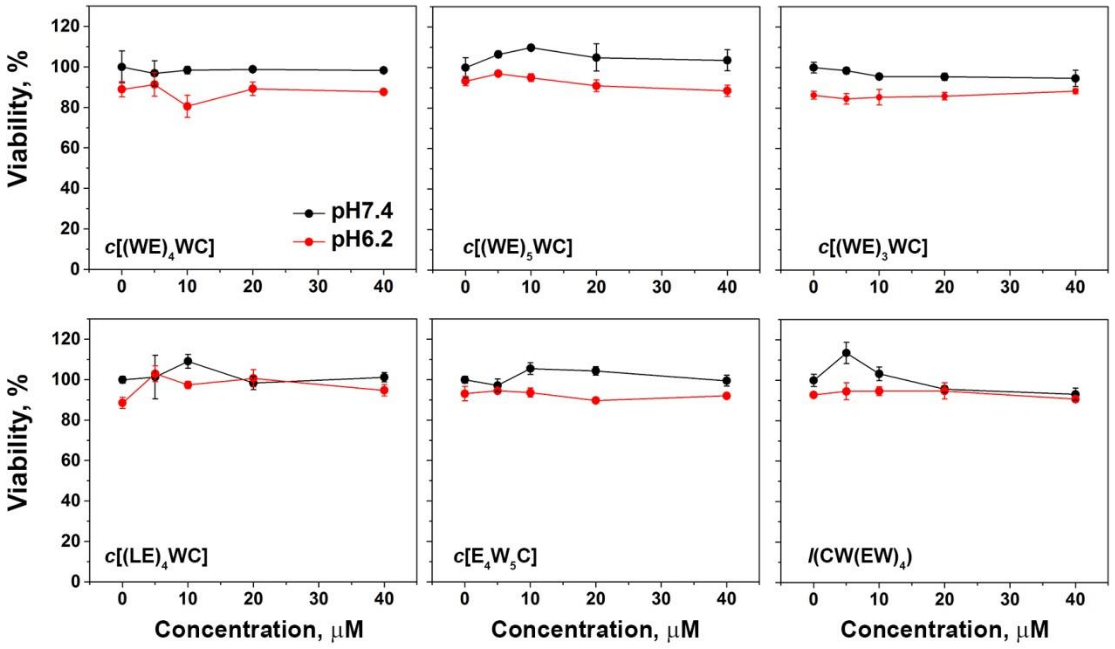

Figure S3. HeLa cells were treated with increasing concentrations of the peptides without FBS at pH 6.2 (red lines and circles) or pH 7.4 (black lines and circles). The same volume of DMEM medium supplemented with 20% FBS, pH 7.4 was added after 2 hours of treatment. After 48 hours of incubation of cells with the peptides the MTS assay was performed to access cell viability.

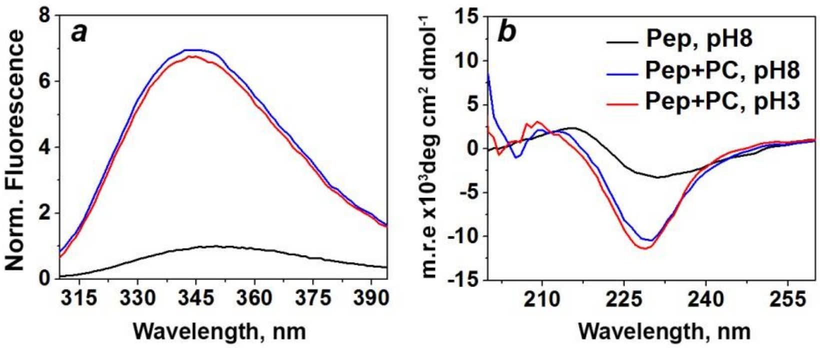

Figure S4. The fluorescence (***a***) and CD (***b***) of the positively-charged asymetric cyclic peptide, *c*[R_4W_5C], in phosphate buffer at pH 8 (black lines) and in the presence of POPC liposomes at pH 8 (blue lines) and pH 3 (red lines) are shown, the excitation wavelength was 280 nm.

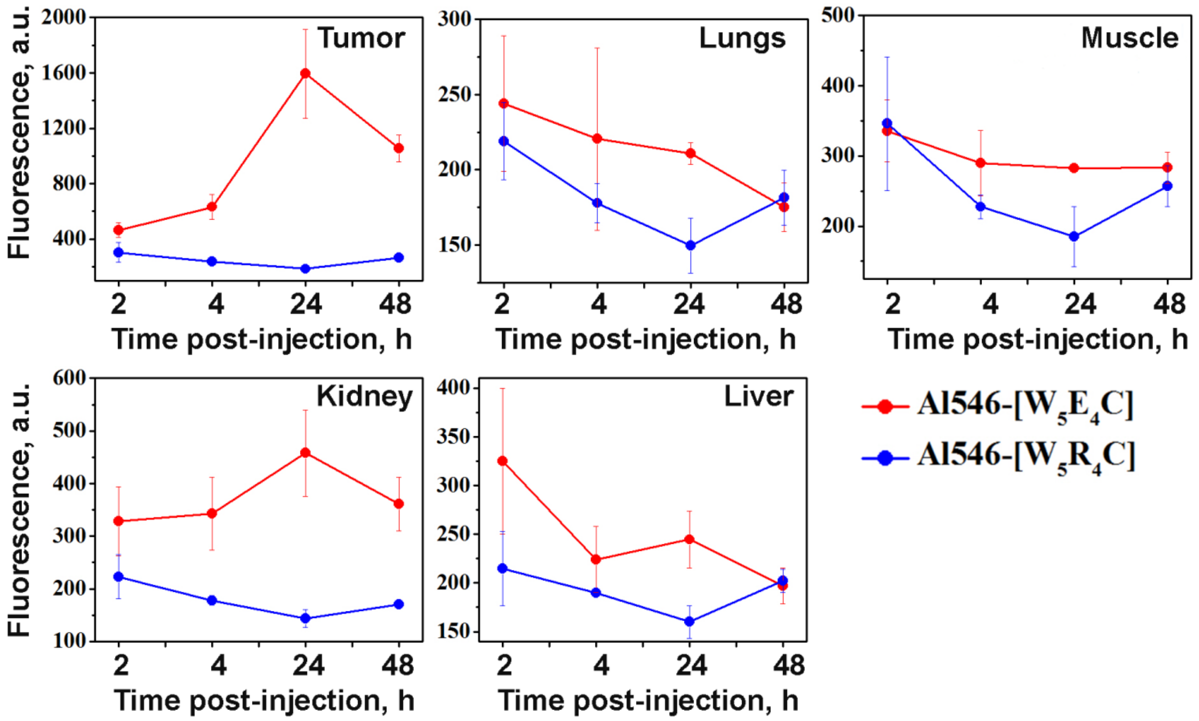

Figure S5. Kinetics of the fluorescent signal changes in tumor, lungs, muscle, kidney and liver at different time points after intravenous administration of the pH-sensitive, *c*[E_4W_5C], and the pH-insensitive, *c*[R_4W_5C], cyclic peptides. This is a supplementary data to Figure 7a and b.

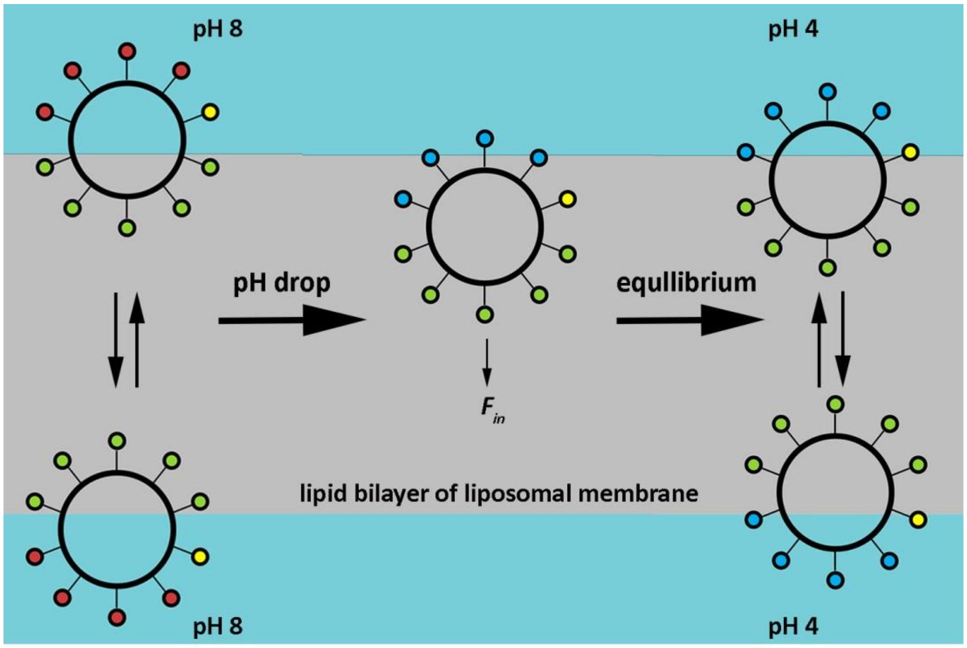

Figure S6. The peptides distribution between outer and inner leaflets of the lipid bilayer in liposomes. At neutral and high pHs, Glu residues are negatively-charged (red circles). Trp residues (green circles) interact with the polar headgroups. Cys residue (yellow circle) could be directed into the bilayer or away depending on cargo hydrophobicity conjugated with Cys. Drop of a pH leads to the protonation of Glu residues (blue circles), which enhances peptides hydrophobicity and induces partition into the bilayer. In the case of liposomes, pH equilibrates fast between inner and outer space, thus the same pH will be outside and inside of a liposome. It would lead to the equilibration of the concentration of cyclic peptides with protonated Glu residues between inner and outer leaflets of the bilayer.

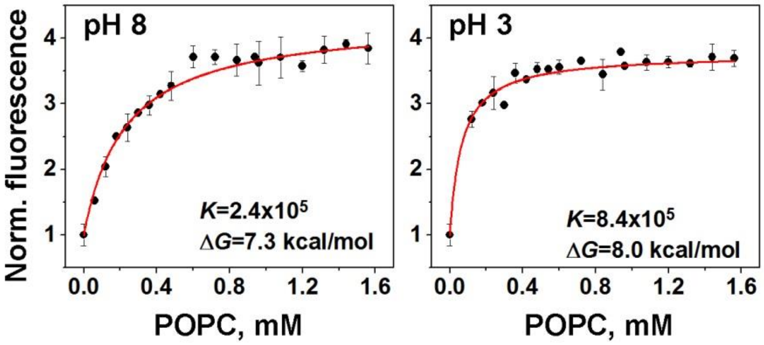

Figure S7. The partition of the asymmetric cyclic $c[E_4W_5C]$ peptide to the lipid bilayer of POPC liposomes was investigated at high and low pHs. Nonlinear least squares curve fitting was performed using Levenberg-Marquardt algorithm.
